# Prevalence and Factors Associated with Caregivers’ Hesitancy in Immunizing Dependent Older Adults with COVID-19 Vaccines: A Cross-Sectional Survey

**DOI:** 10.3390/vaccines10101748

**Published:** 2022-10-19

**Authors:** Saran Thanapluetiwong, Sirintorn Chansirikarnjana, Piangporn Charernwat, Krittika Saranburut, Pichai Ittasakul

**Affiliations:** 1 Division of Geriatric Medicine, Department of Medicine, Faculty of Medicine, Ramathibodi Hospital, Mahidol University, Bangkok 10400, Thailand; 2 Cardiovascular and Metabolic Center, Faculty of Medicine, Ramathibodi Hospital, Mahidol University, Bangkok 10400, Thailand; 3 Department of Psychiatry, Faculty of Medicine, Ramathibodi Hospital, Mahidol University, Bangkok 10400, Thailand

**Keywords:** vaccine hesitancy, COVID-19 vaccine, caregiver, dependent, older adult

## Abstract

Background: Coronavirus disease 2019 (COVID-19) vaccinations have been proven to prevent hospitalization and mortality. However, some caregivers may be hesitant to authorize COVID-19 vaccination of people under their care. Our study aimed to evaluate factors associated with caregiver hesitancy to authorize vaccination of dependent older adults. Method: We conducted a cross-sectional telephone survey of vaccine hesitancy among caregivers of dependent older patients in the geriatric clinic of Ramathibodi Hospital. Caregivers were contacted and interviewed by trained interviewers from 20 June to 25 July 2021. Results: The study enrolled 318 participants with a mean age of 55.9 years. The majority of the participants were the patients’ children (86.5%). In total, 39.9% of participants were hesitant to authorize COVID-19 vaccination of the older adults under their care. Factors associated with caregiver vaccine hesitation were uneasiness, anxiety, agitation, sadness, and worry in association with social distancing, refusal to receive a COVID-19 vaccine, and concern about vaccine manufacturers. Conclusion: The prevalence of caregiver hesitancy to allow older adults to undergo COVID-19 vaccination was relatively high, and several factors associated with this vaccine hesitancy were identified. These findings may aid efforts toward COVID-19 vaccination of dependent older adults.

## 1. Introduction

The coronavirus disease 2019 (COVID-19) pandemic began in January 2019 [1], and over 400 million people worldwide had been infected by March 2022 [2]. Some of those people developed the severe acute respiratory syndrome coronavirus 2 (SARS-CoV-2) infections, leading to high morbidity and mortality rates. In Thailand, there have been 2.9 million confirmed COVID-19 cases and over 22,000 deaths as of 2 March 2022 [3]. The elderly are among the most vulnerable groups in terms of SARS-CoV-2 infection [4,5,6,7]. According to a report by the World Health Organization (WHO), the COVID-19 mortality rate of older people in Thailand was 7.4%, compared with 0.98% for the general population [8].

COVID-19 vaccinations can prevent infection, hospitalization, and mortality [9,10,11]. However, as SARS-CoV-2 evolved over time, from the wild-type to the now-predominant Omicron variant [12], COVID-19 vaccine effectiveness seemed to wane, and booster doses were required [13,14]. Despite the efficacy of the vaccines and the higher mortality rates of COVID-19, some older adults were still hesitant to receive one. The Strategic Advisory Group of Experts on Immunization of the WHO defined vaccine hesitancy as a delay in acceptance of vaccination, or refusal of vaccination despite the availability of vaccination services [15,16]. One systematic review and meta-analysis reported prevalence rates of unwillingness and uncertainty to receive a COVID-19 vaccine of 27.0% and 19.3%, respectively, in older adults. In a US study, factors associated with vaccine unwillingness were a low income, low level of education, and Hispanic ethnicity [17].

In our previous study, the prevalence of COVID-19 vaccine hesitancy among Thai seniors was relatively high; 44.3% of this group were hesitant to get the COVID-19 vaccine [18]. In our geriatric clinic, it was observed that some older patients depend on their caregivers due to underlying conditions. Thus, they lack the capacity to make their own vaccination decisions. In such cases, the caregivers, who are typically family members, need to make the decision regarding COVID-19 vaccination on behalf of the patient [19]. However, some caregivers refuse to authorize COVID-19 vaccination of patients under their supervision. The goal of this study is to determine the factors that contribute to hesitancy among caregivers to authorize vaccination for the dependent older adults under their care. The findings of this study could improve COVID-19 vaccination rates among dependent older adults.

## 2. Materials and Methods

### 2.1. Setting and Study Design

The Human Research Ethics Committee of the Faculty of Medicine, Ramathibodi Hospital, Mahidol University (COA. No. MURA2021/1063) approved the study protocol. We conducted a cross-sectional telephone survey of vaccine hesitancy (as defined above) among caregivers of older patients in the geriatric clinic of Ramathibodi Hospital, which provides tertiary care for this group. Patients aged ≥ 60 years who visited a geriatric clinic in the past 2 years were identified in the hospital database. Patients who were independent and could make their own decisions to undergo COVID-19 vaccination were excluded from the study. The dependent patients were older patients with physical and mental dependence, as well as cognitive impairment. Caregivers of dependent patients who identified themselves as the patients’ representatives, responsible for making COVID-19 vaccination decisions on their behalf, were invited to take part in this study. All participants provided verbal informed consent, which was obtained according to the approved verbal informed consent protocol of the Human Research Ethics Committee of the Faculty of Medicine, Ramathibodi Hospital, Mahidol University. We did not obtain written informed consent because we conducted a telephone survey, and it was inconvenient for participants to sign written informed consent forms and handle documents during the pandemic. The consenting participants were interviewed by a trained interviewer. The survey was performed by telephone from 20 June to 25 July 2021. The study was conducted according to the Declaration of Helsinki and Good Clinical Practice guidelines [20].

### 2.2. Questionnaire

The questionnaire used in this study was developed after a review of the literature [21,22,23,24,25,26,27,28,29,30,31,32,33]. A consensus was reached among experts, including psychiatrists and geriatricians. The questionnaire was divided into five sections: caregiver sociodemographic data, patient sociodemographic data, medical history, COVID-19 pandemic-related information, and COVID-19 vaccine-related information. A pilot study (*n* = 10) was performed to improve the linguistic clarity of the survey items. The pilot data were not included in any subsequent analyses. The final version of the questionnaire typically required 30–45 min to complete. The questionnaire was originally developed in the Thai language.

#### 2.2.1. Caregiver Sociodemographic Characteristics

Participants were asked about their sociodemographic characteristics, including age, gender, marital status, education, relationship with patient, employment status, monthly income, income loss due to COVID-19, and vaccination history [including the influenza, pneumococcal, zoster, and diphtheria-tetanus-pertussis (DTP) vaccines].

#### 2.2.2. Patient Sociodemographic Characteristics and Medical History

The sociodemographic characteristics of the patients were collected in a similar manner as for the caregivers. In addition, the participants were asked to report the patients’ medical history, including body mass index (BMI), ambulation, hearing problems, visual problems, history of smoking and alcohol drinking, food and drug allergies, underlying diseases, cognitive complaints, hospitalization in the previous year, and perceived overall health status.

#### 2.2.3. COVID-19 Pandemic-Related Information

Participants were questioned regarding their general knowledge of COVID-19, their primary source of information on COVID-19, confidence in governmental and public health agency information about COVID-19, confidence regarding the capacity of Thailand’s healthcare system to care for COVID-19 patients, confidence regarding governmental measures to control COVID-19 infection, self-perceived risk of being infected with COVID-19, self-perceived risk of developing a severe COVID-19 infection, attitudes toward social distancing, and intention to be vaccinated against COVID-19.

#### 2.2.4. COVID-19 Vaccine-Related Information

Participants were questioned regarding their hesitancy to authorize COVID-19 vaccination of the patients under their care. They were asked if they knew people who had had a severe reaction to the COVID-19 vaccine, whether they intended to be vaccinated themselves, whether they had already received a COVID-19 vaccination, and whether they wanted those under their care to be vaccinated for COVID-19. They were also asked if they based their decision regarding their elderly dependents getting vaccinated on the manufacturer of the vaccine. Finally, the participants were also asked if they would still want their patients to receive a vaccination if the manufacturer was not that highly anticipated.

Respondents who were hesitant to allow their patients to receive a COVID-19 vaccine were questioned as to why that was the case, as were those willing to authorize vaccination.

### 2.3. Statistical Analysis

Nominal data, such as the presence of underlying disorders, are summarized as numbers and percentages of patients. Depending on the normality of the data distribution, continuous variables such as age are summarized as mean ± standard deviation (SD). To analyze categorical variables, the chi-square test or Fisher’s exact test was used, while an independent *t* test was used for continuous variables. Binary logistic regression was used to identify influencing factors. Only statistically significant factors in the univariable logistic regression model were included in the multivariable logistic regression model. SPSS for Windows software (ver. 26.0; IBM Corp., Armonk, NY, USA) was used for all statistical analyses. Statistical significance was defined as a *p* value < 0.05.

## 3. Results

**Figure 1:**
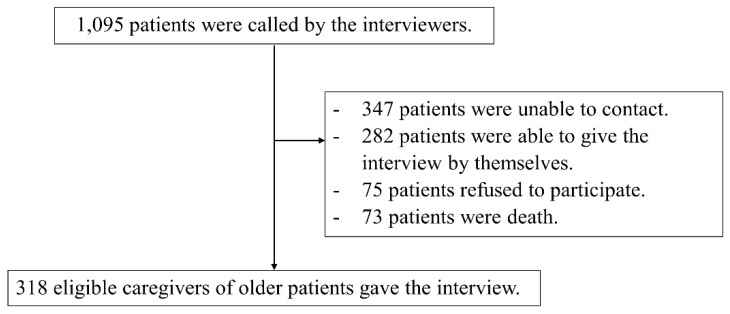
Study flow diagram.

Of the 1095 patients contacted, 318 (29.0%) had caregivers who declared themselves as the patients’ representatives; these caregivers were enrolled in the study (Figure 1). Among the 318 participants, 127 (39.9%) were hesitant to authorize COVID-19 vaccination for the dependent older adults under their care, whereas 191 (60.1%) showed no hesitancy.

### 3.1. Sociodemographic Characteristics

The sociodemographic data of the caregivers are shown in Table 1 and Table 2. The participants ranged in age from 26 to 91 years (mean ± SD age = 55.9 ± 11.5 years; age information was provided by 313 caregivers). Most of the caregivers were female (76.4%) and married (53.8%), with a bachelor’s degree or higher (87.1%). In total 86.5% of the caregivers were the children of the patients, while 9.1% were spouses and 4.4% were siblings.

### 3.2. Sociodemographic Characteristics and Medical History of Dependent Older Adults

The sociodemographic data of the dependent older patients are shown in Table 3 and Table 4. The mean ± SD age was 83.8 ± 8.4 years (range: 60–107 years). Most of the patients were female (73.6%) and of Thai ethnicity (93.1%). In total, 45.0% were married and 63.2% lived in Bangkok. Regarding health status, 19.2% of the patients were bedbound and 10.1% depended on tube feeding. Moreover, 84.6% of the patients had cognitive complaints, 61.0% were diagnosed with dementia, 31.4% had experienced a fall, and 30.2% were admitted to hospital at least once in the previous year.

### 3.3. COVID-19 Pandemic-Related Information

The results for the questionnaire items pertaining to COVID-19 pandemic-related information are provided in Table 5. Most of the participants (52.5%) thought that they knew “quite a lot” or “a lot” about COVID-19, and 43.4% stated that their source of COVID-19 information was television/radio. Caregivers who sometimes felt uneasy, anxious, agitated, sad, or worried when practicing social distancing were more hesitant to authorize COVID-19 vaccination of the dependent older adults under their care [odds ratio (OR) = 2.508; 95% confidence interval (CI): 1.400–4.491, *p* = 0.002] (Table 6).

### 3.4. COVID-19 Vaccine-Related Information

**Figure 2:**
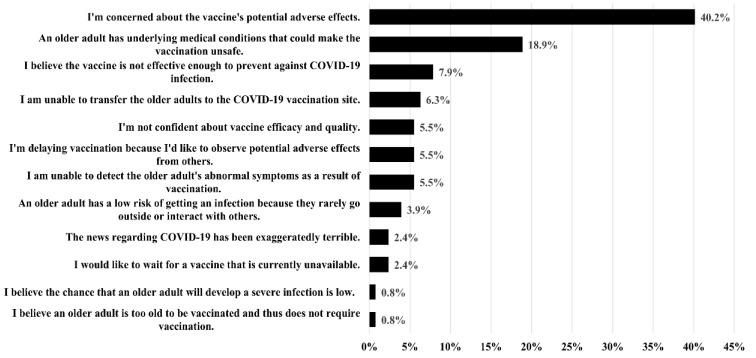
Reasons cited by caregivers for hesitancy to authorize COVID-19 vaccination of the older adults under their care.

**Figure 3:**
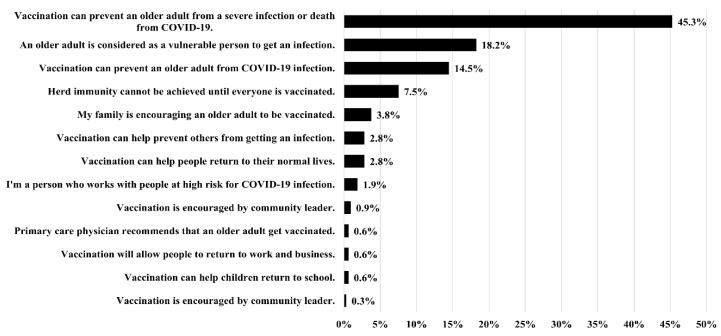
Reasons cited by caregivers for authorizing COVID-19 vaccination of the older adults under their care.

As shown in Table 5, 39.9% of the caregivers were hesitant to authorize COVID-19 vaccination of the older adults under their care, and 13.5% refused to authorize vaccination. In total, 96.2% of the caregivers intended to be vaccinated against COVID-19 themselves, while 76.7% had already been vaccinated. The most common reasons for COVID-19 vaccine hesitancy among the caregivers were concerns regarding adverse effects (40.2%), possible complications caused by an underlying disease (18.9%), and the belief that the vaccines are not effective for preventing COVID-19 infection (7.9%) (Figure 2). The most common reasons for supporting vaccination of older adults were as follows: COVID-19 vaccines can prevent severe infection and death (45.3%); dependent older adults are a vulnerable group (18.2%); and COVID-19 vaccines can prevent COVID-19 infection in older adults (14.5%) (Figure 3). Compared with caregivers exhibiting vaccine acceptance, those who refused to authorize COVID-19 vaccination of the dependent older adults under their care were more likely to show vaccine hesitancy (OR = 3.779; 95% CI: 1.652–8.648, *p* = 0.002). Caregivers who stated that the manufacturer of the COVID-19 did not influence their decision to authorize vaccination were less likely to exhibit vaccine hesitancy (OR = 0.267; 95% CI: 0.152–0.471, *p* < 0.001). 

## 4. Discussion

To our knowledge, this study is the first study to investigate the hesitancy of caregivers to authorize COVID-19 vaccination for the dependent older adults under their care. In total, 318 caregivers were contacted and interviewed. We discovered that 39.9% of the participants were hesitant to allow the older persons under their care to be vaccinated. Caregivers who sometimes felt uneasy, anxious, agitated, sad, or worried when they practiced social distancing were more hesitant to authorize vaccination, as were caregivers who themselves refused COVID-19 vaccination. As expected, an unexpected vaccine manufacturer also contributed to hesitancy. 

The proportion of caregivers hesitant to authorize COVID-19 vaccination of the older adults under their care was high. Practicing social distancing and anxiety have been linked with COVID-19 vaccine hesitancy [34,35,36], as has low compliance with social distancing [34,35]. This is in line with our finding that caregivers with anxiety were more vaccine-hesitant. We also found that COVID-19 vaccine refusal was associated with greater vaccine hesitancy among the caregivers. Caregivers who themselves refused to be vaccinated showed stronger intentions not to authorize vaccination of the older adults under their care, which is a barrier to achieving herd immunity. In some studies, patterns of COVID-19 vaccine hesitancy and refusal/rejection were relatively similar [37,38].

An unexpected vaccine manufacturer was among the factors associated with caregiver hesitation to authorize vaccination. When this study was conducted, Thailand was facing the emerging Delta COVID-19 variant, which caused a surge in cases [39]. Two COVID-19 vaccines were available: the Oxford- ChAdOx1 nCoV-19 vaccine (ChAdOx1 nCoV-19; AstraZeneca) and inactivated SARS-CoV-2 vaccine (CoronaVac). However, only ChAdOx1 nCoV-19 had proven efficacy against the Delta variant [40]. This could explain why the COVID-19 vaccine manufacturer affected the caregivers’ decisions. In this respect, the results of this study were similar to those of our previous study on older adults’ attitudes toward vaccines [18].

A recent systematic review and meta-analysis found that a low income and low levels of education were associated with higher vaccine hesitancy in older adults [17]; however, we could not replicate this finding. This may be explained by the income of most of the participants in our study being higher than the average of Thai people [41]. Furthermore, the majority of our caregivers (87.1%) had a bachelor’s degree or higher. In other words, our participants had a better baseline socioeconomic status than the general Thai population. Moreover, the factors influencing vaccine hesitancy might not be the same between caregivers and dependent older adults. 

The main strength of our study was that it was the first to analyze the attitudes of caregivers toward COVID-19 vaccination of the older adults under their care. Given that caregivers play a crucial role in medical decision-making for dependent older adults, understanding caregiver attitudes is important for promoting COVID-19 vaccination among older adults. Our study also had some limitations. First, we enrolled participants from the hospital database of a geriatric clinic in a university hospital. Thus, the results should be interpreted with caution, especially as some participants refused to provide sensitive information such as their incomes, leading to missing data. Finally, although we demonstrated that various factors were associated with caregiver hesitancy to authorize vaccination of the older adults under their care, we could not demonstrate causality.

## 5. Conclusions

The proportion of caregivers in this study hesitant to authorize vaccination of the older adults under their care was relatively high. Feeling uneasy, anxious, agitated, sad, or worried when practicing social distancing, a refusal to be vaccinated against COVID-19, and an unexpected vaccine manufacturer were all linked to vaccine hesitation among caregivers. These findings may aid efforts to vaccinate dependent older adults against COVID-19. Strategies to help people cope with anxiety, vaccines with high efficacy in terms of preventing infection, and the provision of accurate information regarding the benefits of vaccination are necessary to improve vaccine acceptance. 

## Figures and Tables

**Table 1:** Baseline characteristics of the caregivers (N = 318).

Baseline Characteristics	*n*	%
Age (y) (*n* = 313)		
<40	27	8.5
40–59	160	50.3
≥60	126	39.6
Females	243	76.4
Marital status		
Single	129	40.6
Married	171	53.8
Divorced	5	1.6
Widow	13	4.1
Education level		
Elementary school or lower	12	3.8
High school	29	9.1
Bachelor’s degree or higher	277	87.1
Current residence		
Bangkok	201	63.2
Other province	117	36.8
Relationship with patient		
Spouse	29	9.1
Child	275	86.5
Sibling	14	4.4
Employment status		
Unemployed	54	17
Part-time	65	20.4
Full-time	28	8.8
Retired	171	53.8
Monthly income (baht) (*n* = 239)		
≤10,000	37	15.5
10,001–20,000	50	20.9
20,001–50,000	108	45.2
≥50,001	44	18.4
Income loss due to COVID	95	29.9
History of vaccination		
Influenza vaccine	70	22
Zoster vaccine	295	92.8
Pneumococcal vaccine	276	86.8
DTP vaccine	130	40.9

*n*, number; DTP, diphtheria-tetanus-pertussis.

**Table 2:** Baseline characteristics of caregivers: comparison between the vaccine acceptance and vaccine hesitancy groups.

Characteristics	Acceptance		Hesitancy		χ^2^	*p* Value
	(*n* = 191)		(*n* = 127)			
	*n*	%	N	%		
Age (y) (*n* = 313)						
<40	13	6.90%	14	11.30%	2.56	0.278
40–59	95	50.30%	65	52.40%		
≥60	81	42.90%	45	36.30%		
Female	143	74.90%	100	78.70%	0.634	0.426
Marital status						
Single	73	38.30%	56	44.10%	1.11	0.775
Married	107	56.00%	64	50.40%		
Divorced	3	1.60%	2	1.60%		
Widow	8	4.20%	5	3.90%		
Education level						
Elementary school or lower	6	3.10%	6	4.70%	0.561	0.755
High school	18	9.40%	11	8.70%		
Bachelor’s degree or higher	167	87.40%	110	86.60%		
Current residence						
Bangkok *	129	67.50%	72	56.70%	3.859	0.049
Other province	62	32.50%	55	43.30%		
Relationship with patient						
Spouse	21	11.00%	8	6.30%	2.047	0.359
Child	162	84.80%	113	89.00%		
Sibling	8	4.20%	6	4.70%		
Employment status						
Unemployed	33	17.30%	21	16.50%	0.442	0.932
Part-time	41	21.50%	24	18.90%		
Full-time	16	8.40%	12	9.40%		
Retired	101	52.90%	70	55.10%		
Monthly income (baht) (*n* = 239)						
≤10,000	23	16.10%	14	14.60%	0.362	0.948
10,001–20,000	31	21.70%	19	19.80%		
20,001–50,000	64	44.80%	44	45.80%		
≥50,001	25	17.50%	19	19.90%		
Income loss due to COVID	51	26.70%	44	34.60%	2.298	0.13
History of vaccination						
Influenza vaccine	42	22.00%	28	22.00%	0	0.99
Zoster vaccine	176	92.10%	119	93.70%	0.275	0.6
Pneumococcal vaccine	168	88.00%	108	85.00%	0.567	0.451
DTP vaccine	82	42.90%	48	37.70%	0.833	0.361

N, number; χ^2^, chi-squared; DTP, diphtheria-tetanus-pertussis. * *p* < 0.05.

**Table 3:** Baseline characteristics of the dependent older adults (*n* = 318).

Baseline Characteristics	*n*	%
Age (y)		
60–69	18	5.7
70–79	77	24.2
80–89	144	45.3
≥90	79	24.8
Female	234	73.6
Ethnicity		
Thai	296	93.1
Chinese	22	6.9
Marital status		
Single	19	6
Married	143	45
Divorced	8	2.5
Widow	148	46.5
Children living in the same home	36	11.3
Education		
Elementary school or lower	178	56
High school	61	19.2
Bachelor’s degree or higher	79	24.8
Accommodation		
House/condominium	308	96.9
Nursing home	10	3.1
BMI (*n* = 298)		
<18.5	42	13.2
18.5–22.9	124	39
23–24.9	65	20.4
25–30	58	18.2
>30	9	2.8
Ambulation		
Bedbound	61	19.2
Ambulation	257	80.8
Feeding		
Oral	286	89.9
Tube feeding	32	10.1
Hearing impairment	109	34.3
Visual problems		
Blindness	30	9.4
Visual impairment	74	23.3
Normal	214	67.3
History of smoking	49	15.4
History of alcohol consumption	5	1.6
Food allergy	13	4.1
Drug allergy	88	27.7
History of vaccination		
Influenza vaccine	288	90.6
Zoster vaccine	49	15.4
Pneumococcal vaccine	134	42.1
DTP vaccine	140	44
Underlying disease		
Diabetes	91	28.6
Chronic kidney disease	34	10.7
Respiratory disease	37	11.6
Psychiatric illness	40	12.6
Subjective cognitive complaints	269	84.6
Dementia diagnosis	194	61
History of falls in the past year	100	31.4
Hospitalization in the past year	96	30.2
Perceived overall health status		
Worst/bad	40	12.6
Average	125	39.3
Good/best	153	48.1

*n*, number; BMI, body mass index; DTP, diphtheria-tetanus-pertussis.

**Table 4:** Baseline characteristics of dependent older adults associated with caregiver hesitancy to authorize COVID-19 vaccination.

Characteristics	Acceptance		Hesitancy		χ^2^	*p* Value
	(*n* = 191)		(*n* = 127)			
	*n*	%	*n*	%		
Age (y)						
60–69	11	5.80%	7	5.50%	3.132	0.372
70–79	41	21.50%	36	28.30%		
80–89	86	45.00%	58	45.70%		
≥90	53	27.70%	26	20.50%		
Female	140	73.30%	94	74.00%	0.2	0.887
Ethnicity						
Thai	175	91.60%	121	95.30%	1.58	0.209
Chinese	16	8.40%	6	4.70%		
Marital status						
Single	10	5.20%	9	7.10%	0.846	0.839
Married	87	45.50%	56	44.10%		
Divorced	4	2.10%	4	3.10%		
Widow	90	47.10%	58	45.70%		
Children living in the same home	21	11.00%	15	11.80%	0.051	0.822
Education level						
Elementary school or lower	108	56.50%	70	55.10%	0.227	0.893
High school	35	18.30%	26	20.50%		
Bachelor’s degree or higher	48	25.10%	31	24.40%		
Accommodation						
House/condominium	187	97.90%	121	95.30%	1.733	0.206
Nursing home	4	2.10%	6	4.70%		
BMI (*n* = 298)						
<18.5	25	13.70%	17	14.70%	1.595	0.81
18.5–22.9	75	41.20%	49	42.20%		
23–24.9	40	22.00%	25	21.60%		
25–30	38	20.90%	20	17.20%		
>30	4	2.20%	5	4.30%		
Ambulation						
Bedbound	30	15.70%	31	24.40%	3.727	0.054
Ambulation	161	84.30%	96	75.60%		
Feeding						
Oral	174	91.10%	112	88.20%	0.714	0.398
Tube feeding	17	8.90%	15	11.80%		
Hearing impairment	65	34.00%	44	34.60%	0.013	0.91
Visual problems						
Blindness	17	8.90%	13	10.20%	1.202	0.548
Visual impairment	41	21.50%	33	26.00%		
Normal	133	69.60%	81	63.80%		
History of smoking	28	14.70%	21	16.50%	0.206	0.65
History of alcohol consumption	4	2.10%	1	0.80%	0.842	0.652
Food allergy	8	4.20%	5	3.90%	0.012	0.912
Drug allergy	52	27.20%	36	28.30%	0.048	0.827
History of vaccination						
Influenza vaccine	174	91.10%	114	89.80%	0.159	0.69
Zoster vaccine	34	17.80%	15	11.80%	2.1	0.147
Pneumococcal vaccine	87	45.50%	47	37.00%	2.283	0.131
DTP vaccine	89	46.60%	51	40.20%	1.284	0.257
Underlying disease						
Diabetes	57	29.80%	34	26.80%	0.352	0.553
Chronic kidney disease	23	12.00%	11	8.70%	0.913	0.339
Respiratory disease	22	11.50%	15	11.80%	0.006	0.936
Psychiatric illness	21	11.00%	19	15.00%	1.091	0.296
Subjective cognitive complaints	158	82.70%	111	87.40%	1.281	0.258
Dementia diagnosis	112	58.60%	82	64.60%	1.127	0.288
History of falls in the past year	57	29.80%	43	33.90%	0.57	0.45
Hospitalization in the past year	56	29.30%	40	31.50%	0.171	0.679
Perceived overall health status						
Worst/bad	19	9.90%	21	16.50%	4.737	0.094
Average	72	37.70%	53	41.70%		
Good/best	100	52.40%	53	41.70%		

*n*, number; χ^2^, chi-squared; BMI, body mass index; DTP, diphtheria-tetanus-pertussis.

**Table 5:** Associations of COVID-19 pandemic and vaccine-related information with caregiver hesitancy to authorize COVID-19 vaccination of the dependent older adults under their care.

COVID-19 Pandemic and Vaccine-Related Information	Acceptance		Hesitancy		χ^2^	*p* Value
	(*n* = 191)		(*n* = 127)			
	*n*	%	*n*	%		
How much do you know about COVID-19?						
Nothing	24	12.60%	15	11.80%	0.096	0.992
Little	57	29.80%	39	30.70%		
Quite a lot	100	52.40%	67	52.80%		
A lot	10	5.20%	6	4.70%		
What is your primary source of COVID-19 information?						
Television, radio	81	42.40%	57	44.90%	7.022	0.219
Newspapers	1	0.50%	3	2.40%		
Friends	33	17.30%	16	12.60%		
News websites	24	12.60%	15	11.80%		
Social networks	44	23.00%	35	27.60%		
Other	8	4.20%	1	0.80%		
What is your level of confidence in governmental and public health information on COVID-19?						
Not confident *	24	12.60%	22	17.30%	9.898	0.019
Quite unconfident	35	18.30%	31	24.40%		
Quite confident	106	55.50%	69	54.30%		
Confident	26	13.60%	5	3.90%		
How confident are you in Thailand’s healthcare system’s ability to treat COVID-19 patients?						
Not confident	17	8.90%	12	9.40%	7.647	0.054
Quite unconfident	21	11.00%	28	22.00%		
Quite confident	110	57.60%	65	51.20%		
Confident	43	22.50%	22	17.30%		
How effective are the government’s measures for controlling COVID-19 infection?						
Insufficient	74	38.70%	68	53.50%	6.885	0.076
Somewhat insufficient	71	37.20%	36	28.30%		
Somewhat sufficient	43	22.50%	22	17.30%		
Sufficient	3	1.60%	1	0.80%		
What is your risk of being infected with COVID-19?						
Very low	24	12.60%	11	8.70%	3.974	0.264
Low	89	46.60%	59	46.50%		
High	58	30.40%	35	27.60%		
Very high	20	10.50%	22	17.30%		
What are the chances that you will experience a severe COVID-19 infection or associated life-threatening condition?						
Very low	20	10.50%	5	3.90%	6.933	0.074
Low	84	44.00%	57	44.90%		
High	61	31.90%	38	29.90%		
Very high	26	13.60%	27	21.30%		
Do you feel uneasy/anxious/agitated/sad/worried when you have to practice social distancing?						
Never *	145	75.90%	78	61.40%	7.859	0.049
Sometimes	38	19.90%	42	33.10%		
Often	6	3.10%	5	3.90%		
Always	2	1.00%	2	1.60%		
Do you know anyone who has had a severe reaction to the COVID-19 vaccine?						
No	171	89.50%	107	84.30%	1.932	0.165
Yes	20	10.50%	20	15.70%		
Do you intend to be vaccinated against COVID-19?						
No	4	2.10%	8	6.30%	3.714	0.071
Yes	187	97.90%	119	93.70%		
Have you already been vaccinated against COVID-19?						
No *	37	19.40%	37	29.10%	4.071	0.044
Yes	154	80.60%	90	70.90%		
Do you refuse to authorize COVID-19 vaccination for the older adults under your care?						
No *	175	91.60%	100	78.70%	10.827	0.001
Yes	16	8.40%	27	21.30%		
Did the manufacturer influence your decision to authorize COVID-19 vaccination for the older adults under your care?						
No *	100	52.40%	28	22.00%	29.137	<0.001
Yes	91	47.60%	99	78.00%		
Would you authorize COVID-19 vaccination for the older adults under your care if the manufacturer was different from what you expected?						
No *	33	17.30%	32	25.20%	11.606	0.003
Yes	128	67.00%	61	48.00%		
Unsure	30	15.70%	34	26.80%		

*n*, number; χ^2^, chi-squared. * *p* < 0.05.

**Table 6:** Results of logistic regression analysis of caregiver hesitancy to authorize COVID-19 vaccination of the dependent older adults under their care.

Variable	Univariate			Multivariate		
	OR	95% CI	*p* Value	aOR	95% CI	*p* Value
Current residential area						
Bangkok	Ref					
Other province	1.589	1.000–2.527	0.05	1.476	0.877–2.486	0.143
What is your level of confidence in governmental and public health information on COVID-19?						
Not confident	Ref					
Quite unconfident	0.966	0.455–2.053	0.929	1.126	0.483–2.627	0.784
Quite confident	0.71	0.370–1.365	0.304	1.26	0.598–2.656	0.543
Confident	0.21	0.069–0.642	0.006	0.374	0.111–1.258	0.112
Do you feel uneasy/anxious/agitated/sad/worried when you have to practice social distancing?						
Never	Ref					
Sometimes *	2.055	1.224–3.449	0.006	2.508	1.400–4.491	0.002
Often	1.549	0.458–5.238	0.481	1.54	0.392–6.048	0.536
Always	1.859	0.257–13.453	0.539	1.331	0.176–10.083	0.782
Have you already had a COVID-19 vaccination?						
No	1.711	1.013–2.892	0.045	1.287	0.697–2.376	0.419
Yes	Ref					
Do you refuse to authorize COVID-19 vaccination for the older adults under your care?						
No	Ref					
Yes *	2.953	1.518–5.745	0.001	3.779	1.652–8.648	0.002
Did the manufacturer influence your decision to authorize COVID-19 vaccination for the older adults under your care? *						
No *	0.257	0.155–0.427	<0.001	0.267	0.152–0.471	<0.001
Yes	Ref					
Would you authorize COVID-19 vaccination for the older adults under your care if the manufacturer was different from what you expected?						
No	2.035	1.146–3.612	0.015	1.04	0.516–2.096	0.913
Yes	Ref					
Unsure	2.378	1.334–4.239	0.003	1.248	0.637–2.446	0.518

OR, odds ratio; aOR, adjusted odds ratio; CI, confidence interval; Ref, reference group. * *p* < 0.05.

## Data Availability

The datasets used and analyzed during the current study are available from the corresponding author upon reasonable request.
